# Desmoplastic small round cell tumour: Cytological and immunocytochemical features

**DOI:** 10.1186/1742-6413-2-6

**Published:** 2005-03-18

**Authors:** Nara M Granja, Maria D Begnami, Jeni Bortolan, Adhemar Longatto Filho, Fernando C Schmitt

**Affiliations:** 1 Pathology Department, School of Medicine, São Paulo University, São Paulo, Brazil; 2 Department of Pathology and Treatment and Research Center of A.C. Camargo Hospital, São Paulo, Brazil; 3 Life and Health Sciences Research Institute, Health Sciences School, University of Minho, Braga, Portugal; 4 Pathology Division, Adolfo Lutz Institute, São Paulo, Brazil; 5 Medical Faculty, Department of Pathology, University of Porto, Porto, Portugal; 6 IPATIMUP – Institute of Molecular Pathology and Immunology of University of Porto, Porto, Portugal

## Abstract

**Background:**

Desmoplastic small round cell tumor (DSRCT) is a rare and highly aggressive neoplasm. The cytological diagnosis of these tumors can be difficult because they show morphological features quite similar to other small round blue cells tumors. We described four cases of DSRCT with cytological sampling: one obtained by fine needle aspiration biopsy (FNAB) and three from serous effusions. The corresponding immunocytochemical panel was also reviewed.

**Methods:**

Papanicolaou stained samples from FNAB and effusions were morphologically described. Immunoreaction with WT1 antibody was performed in all cytological samples. An immunohistochemical panel including the following antibodies was performed in the corresponding biopsies: 34BE12, AE1/AE3, Chromogranin A, CK20, CK7, CK8, Desmin, EMA, NSE, Vimentin and WT1.

**Results:**

The smears showed high cellularity with minor size alteration. Nuclei were round to oval, some of them with inconspicuous nucleoli. Tumor cells are clustered, showing rosette-like feature. Tumor cells in effusions and FNA were positive to WT1 in 3 of 4 cytology specimens (2 out 3 effusions and one FNA). Immunohistochemical reactions for vimentin, NSE, AE1/AE3 and WT1 were positive in all cases in tissue sections.

**Conclusion:**

The use of an adjunct immunocytochemical panel coupled with the cytomorphological characteristics allows the diagnosis of DSRCT in cytological specimens.

## Introduction

Desmoplastic small round cell tumor (DSRCT) is a rare and highly aggressive neoplasm described as a distinct clinicopathologic entity in 1989 by Gerald and Rosai [1]. Usually affecting young males and presenting as an abdominal mass, the tumor grows along serosal membranes with multiple nodules attached to the peritoneal surface [2]. Other primary sites have been reported as pleura [3], paratesticular region [4], bone and soft tissues [5] and ovary [6,7].

Histologically, a typical feature of DSRCT is the presence of clusters of tumor cells distributed within a cellular stroma. The shape of clusters varies from round to elongate. Tumor cells are small to medium-sized with round to oval hyperchromatic nuclei, with inconspicuous nucleoli. Necrotic cells and mitosis are common features. Cytoplasm is usually scanty, and cell borders are indistinct. Intracytoplasmic eosinophilic rhabdoid inclusions may be found in larger cells with nuclear pleomorphism [8]. The immunohistochemical profile shows divergent differentiation, a striking feature of this tumor. DSRCT may present a problem in the differential diagnosis with other round cell tumors. Tumor cells are immunoreactive for epithelial, neural and myogenic markers [2]. Cytogenetical studies have demonstrated a reciprocal chromosome translocation between the Ewing's sarcoma gene on chromosome 22 and the Wilms' tumour gene WT1 on chromosome 11, which is distinct from the translocation observed in Ewing sarcoma/peripheral neuroectodermal tumor (PNET) [9].

The cytological smears of DSRCT obtained by FNAB are moderately cellular. Tumor cells show round to oval nuclei with fine chromatin and inconspicuous nucleoli. Cytoplasm is scanty to moderate, with variable number of vacuoles. Tumor cells are arranged in loose clusters. Occasionally, spindle fibroblast-like cells are observed. Stromal fragments may be detected [10]. Effusion samples show cohesive cell clusters and similar cytological features. Mitoses or individual necrotic cells may be present, as nuclear molding [3]. In the current study, we describe the morphological and immunocytochemical features of four cytologic specimens, one of them obtained by FNAB and three from serous effusions (2 peritoneal fluid samples and one pleural effusion), from 3 patients with a diagnosis of DSRCT.

## Materials and methods

We retrieved from the cytological files of Hospital do Cancer – A. C. Camargo four cytological specimens from 3 patients diagnosed with DSRCT, including one fine-needle aspiration sample and 3 fluid samples, during the last five years (2000–2004). FNA was performed on an inguinal mass of one patient. Alcohol-fixed smears were stained with Papanicolaou technique. Serous effusions were prepared with Cytospin (Shandon, Pittsburgh, Pennsylvania, USA). We evaluated two peritoneal fluid samples and one pleural fluid sample. One case (patient 1, peritoneal fluid) had a cellblock available. All cases were confirmed by histological analysis and immunohistochemical reactions. The histological sections were cut in sections of 4 μm and stained with H&E and immunohistochemistry. Immunocytochemical study was also performed on all cases.

Immunohistochemical and immunocytochemical reactions were performed using streptavidin-biotin peroxidase technique with positive and negative controls. Diaminobenzidine was the chromogen. Table 1 shows the antibodies used and dilutions. All antibodies were from DAKO Corporation, Capinteria, CA, U.S.A.

**Table 1:** Antibodies and dilutions used in this study

**Marker**	**Antibody clone**	**Dilution**
34BE12	34BE12	1:100
AE1/AE3	AE1/AE3	1:500
Chromogranin A	DAK-A3	1:100
CK20	KS20.8	1:50
CK7	OV-TL 12/30	1:100
CK8	35BH11	1:100
Desmin	D33	1:100
EMA	E29	1:2000
NSE	BBS/NC/V1-H14	1:1500
Vimentin	Vim 3B4	1:200
WT1	6F-H2	1:400

### Cases

#### Patient 1

22-year-old white female, with abdominal pain. Video-laparoscopy showed a liver mass and multiple peritoneal implants diagnosed as DSRCT. Six months after the diagnosis, she started chemotherapy for four months, and reduction of tumor mass was observed. One month after the end of chemotherapy, the tumor was removed. Macroscopically, tumor mass measured 5.0 × 4.0 × 3.8 cm and was involving uterus, pericolic tissue, and vagina. Histological analysis shows also involvement of both ovaries and large bowel wall. Ten out of 13 lymph nodes showed metastasis of DSRCT. The peritoneal fluid colleted during surgery was negative for neoplastic cells. Eight months after the first surgery, she presented with a recurrence in the abdominal cavity and a new resection of the tumor mass showed involvement of cecal appendix. Peritoneal fluid sample collected at that time was positive for malignant cells. In the follow up examination, seven months after the second surgery, it was found an inguinal tumor mass of 15 mm. FNA was performed and showed DSRCT metastasis. After the diagnosis, this patient was transferred to another institution.

#### Patient 2

Seven year-old male with back pain and fever. CT scan showed pleural effusion and a mediastinal mass measuring 16.0 × 9.0 cm. Tumour mass showed involvement of soft tissues. Surgical biopsy and pleural drainage were performed. The patient was treated with radiotherapy and chemotherapy, but died 8 months after the diagnosis.

#### Patient 3

Male, 15-year-old had acute abdominal pain and was submitted to an exploratory laparotomy that disclosed a large pelvic mass, involving epiplon and sigmoid, cecum, liver and peri-aortic lymph nodes. This patient had multiple nodules on peritoneal surface. The biopsy of tumor was performed. One month after the diagnosis, chemotherapy was initiated. The patient was submitted to chemotherapy during 8 months, with reduction of more than 50% of tumor mass. A second laparotomy was done to excise retroperitoneal and retrovesical mass. At this time peritoneal fluid sample was collected. After surgery, chemotherapy was continued. The patient is alive, with residual disease.

## Results

### Cytological findings

#### Case 1 (Fine needle aspiration)

**Figure 1:**
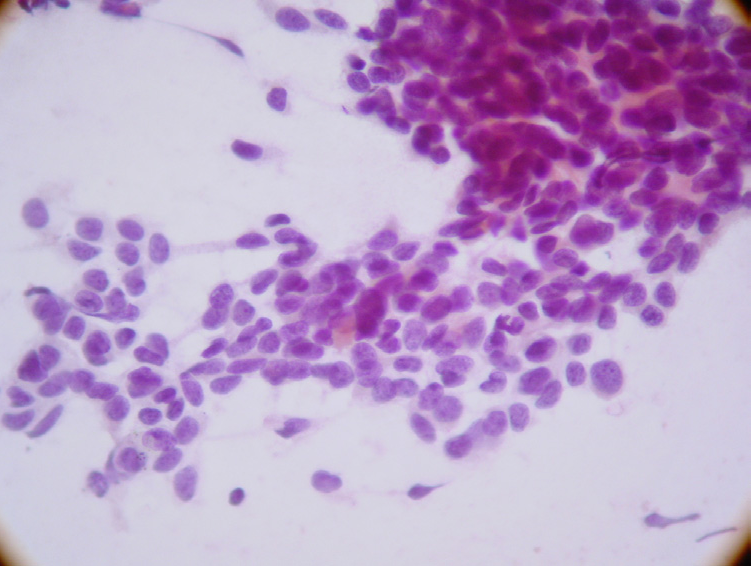
Clusters of small round tumor cells showing rosette-like features in smear of fine needle aspiration specimen of DSRCT.

The smears showed high cellularity. The tumor cells exhibited a slight variation in size. Nuclei were round to oval, some of them with small nucleoli. The cytoplasm was scanty. Tumor cells are clustered, with rare clusters showing rosette-like features. The background of the smears showed lymphocytes. (Figure 1).

#### Cases 1, 2 and 3

**Figure 2:**
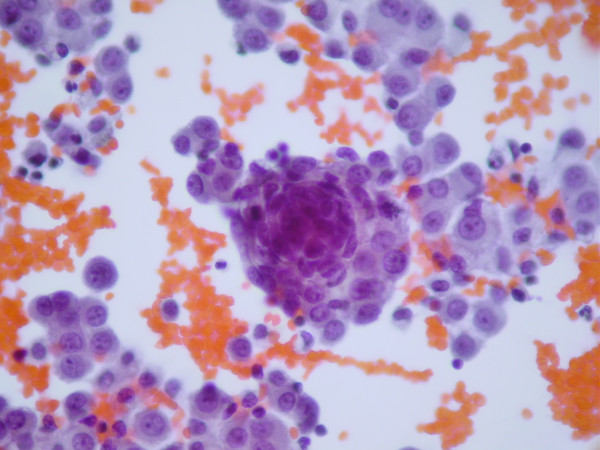
Effusion from patient with DSRCT exhibiting high cellularity. Observe tridimentional clusters of neoplastic cells. Nuclei were round to oval, some of them with small nucleoli and the cytoplasm is scanty.

All fluid samples showed similar features. The samples showed high cellularity. Tumor cells were more frequently arranged in tridimentional clusters, but occasionally, isolated cells are also seen. Additionally, clusters showing rosette-like features are rarely observed. Nuclei were round to oval, some of them with small nucleoli. The cytoplasm was scanty (Figure 2).

### Immunohistochemical and Immunocytochemical findings

**Figure 3:**
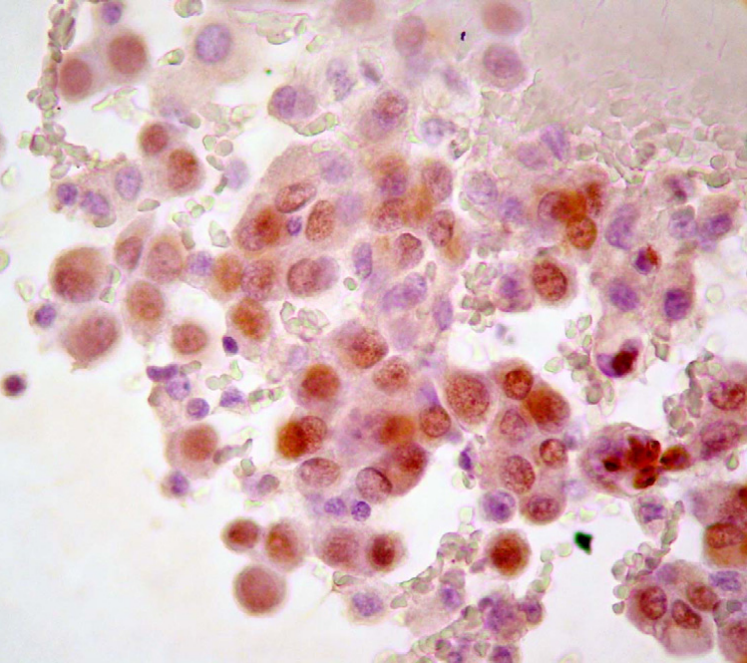
DSRCT tumor cells in effusion showing nuclear positive reaction to WT1.

The distribution of immunoreactivity in histological and cytological samples from the patients are summarized in Table 2. Tumor cells in effusions from patients 1 and 2 and, the smear obtained by FNA (Patient 1) were positive to WT1 (Figure 3).

**Table 2:** Distribution of immunoreactions in patients 1, 2 and 3 histological and cytological samples.

**Marker**	**Patient 1 Biopsy**	**Patient 2 Biopsy**	**Patient 3 Biopsy**	**Patient 1 Cytology**	**Patient 2 Cytology**	**Patient 3 Cytology**
34BE12	-	-	ND	ND	ND	ND
AE1/AE3	+	+	+	ND	ND	ND
Chromogranin A	+	+	ND	ND	ND	ND
CK20	-	-	ND	ND	ND	ND
CK7	-	-	ND	ND	ND	ND
CK8	+	-	ND	ND	ND	ND
Desmin	+	-	ND	ND	ND	ND
EMA	+	+	ND	ND	ND	ND
NSE	+	+	+	ND	ND	ND
Vimentin	+	+	+	ND	ND	ND
WT1	+	+	+	+	+	-
				(Effusion & FNA)		

N.D. = not done.

#### Patient 1

the histological sample collected before chemotherapy, exhibited immunohistochemical positivity for vimentin, epithelial membrane antigen (EMA), neuron specific enolase (NSE), chomogranin A, and desmin in dot-like perinuclear pattern. Cytokeratin expression was observed with anti-cytokeratin cocktail (AE1/AE3) and Cytokeratin 8. Tumor cells also expressed WT1 protein.

#### Patient 2

tumor cells exhibited positivity for vimentin, EMA, NSE, chomogranin A, AE1/AE3 and WT1. Desmin and cytokeratins 7, 20 and 34BE12 were negative.

#### Patient 3

tumor cells exhibited positivity for vimentin, NSE, AE1/AE3 and WT1. The immunohistochemical study was performed before chemotherapy.

## Discussion

DSRCT is a rare neoplasm that affects young patients. It may present a problem in the differential diagnosis with other small round cell tumors. The diagnosis of DSRCT however can be established with correlation of clinical, cytological and immunocytochemical features. The cytological features that we found in the smears obtained by FNA are similar to other descriptions in the literature. Similar to reports of Zeppa et al [11], we did not detect in our smears fragments of fibrosis or cytoplasmic granules or vacuoles. The finding of stromal fragments, frequently seen in FNA is not a common finding in liquid based preparations [12].

One of the characteristics of DSRCTs is its dissemination along serous surfaces. Due to this fact, development of serous effusions is a common clinical finding in DSRCTs patients, with detection of tumor cells in the fluid. In effusions, tumor cells may be present in aggregates but no obviously architectural arrangement is seem.

Demonstration of a divergent phenotype and the reciprocal translocation characteristic of DSRCT are critical to the diagnosis.

In a reported series of 32 cases of DSRCTs [13], 88% of cases were immunoreactive for AE1/AE3, 84% for NSE, 81% for desmin. These results were similar to other previous studies [2]. Lae et al [13] detected positivity to WT1 antibody in 29 out of 32 cases. Our immunohistochemical results are in agreement with other previous studies. Strong membrane expression of HER2/neu and immunoreactivity to c-kit protein are not common findings [14].

The establishment of a specific reciprocal translocation t (11; 22)(p13;12) as diagnostic in DSRCT was based on the results of Sawyer et al [9]. Shen et al [15] and Roberts et al [16] described variants of with other chromosome involved in addition to chromosome 11 and 22. The translocation t (11; 22)(p13;12) involve the EWS gene in 22q24 and WT1 gene in 11p13. This translocation produces the chimeric transcript EWS/WT1 and the related WT1 protein, which can be detected by immunohistochemical method.

EWS gene encodes a protein which the precise function and normal role has not yet been elucidated. Recently, Thomas et al [17] proposed that the protein product of the EWS gene interacts with Brn-3a cellular transcription factor via a direct protein-protein interaction. Native WT1 protein function has not completely known, but it represses transcription in vitro of many genes. WT1 is a tumor-suppressor gene that encodes a protein, which mediates transcriptional repression and interacts with p53 protein [18], product of another tumor suppressor gene, TP53, frequently deleted or mutated in many human tumors. In absence of intact p53 protein, WT1 acts as a transcriptional activator [19]. Normal WT1 protein is expressed in tissues, which undergo mesenchymal-epithelial conversion derived from mesoderm, in a specific period of development [20] and it plays a role in mesothelial formation in embryonic development [21]. Immunohistochemical detection of WT1 in DSRCTs is predictive of the translocation and it also demonstrates that the chimeric protein is expressed in significant amount in tumour cells ^22, 23^. In addiction to consistent WT1 expression, the typical serosal involvement in DSRCT has raised the possibility that this tumor might be a blastematous tumour derived of primitive mesothelium [24]. Mesothelin is a glycoprotein of unknown function strongly expressed in mesothelial cells. Although lack of specificity of expression of mesothelin for mesothelial origin, the expression of this protein in DSRCT may have some significance on histogenisis of this tumor [25].

We detected WT1 immunoreactivity in all tumors tissues and in 2 out of 3 serous effusions with malignant cells, as well as on FNAB smears. The high frequency of DSRCTs with WT1 protein expression suggests that in consensus with clinical tomographic and cytological findings, this antibody may be used to confirm the diagnosis of DSRCT in cytological samples. We observed a negative WT1 reaction in the cytological sample of patient 3. This sample was collected 10 months after the end of chemotherapy protocol. We can hypothesize if chemotherapy hampered a different antigenic pattern in malignant cells, and influenced this result.

Among other small round cell tumors, most of cases of rhabdomyosarcomas and neuroblastomas do not disclose nuclear WT1 staining [26,27]. Comparing DSRCT and Ewing Sarcoma/PNET, Hill et al. [28] detected WT1 nuclear immunoreactivity in all 13 DSRCT cases studied; conversely, all 11 cases of Ewing Sarcoma/PNET were negative. Additionally, Wilm's tumor was demonstrated to present a high percentage of cases with nuclear WT1 staining; for this reason, correlation with clinical findings is necessary to do a differential diagnosis between Wilm's tumour and DSCRT in effusions [26]. On the other hand, it is important to emphasize that malignant mesothelioma should also be considered in the differential diagnosis, since it can show varied histological appearances including sarcomatoid differentiation with desmoplastic areas, or even resembling undifferentiated sarcomas [29]. WT1 might also decorate nuclei of both epithelioid or biphasic mesothelioma but in general, WT1 stain most frequently epithelioid mesotheliomas [30]. The use of a panel of markers can also help in the differential diagnosis.

In conclusion, cytological and immunophenotypical findings in an appropriate clinical context is sufficient to suggest DRSTC, what sounds highly contributive for us, considering the high aggressiveness of this tumor.
